# Calcium-deficiency assessment and biomarker identification by an integrated urinary metabonomics analysis

**DOI:** 10.1186/1741-7015-11-86

**Published:** 2013-03-28

**Authors:** Maoqing Wang, Xue Yang, Fan Wang, Ran Li, Hua Ning, Lixin Na, Yifan Huang, Yue Song, Liyan Liu, Hongzhi Pan, Qiuju Zhang, Lijun Fan, Ying Li, Changhao Sun

**Affiliations:** 1 Department of Nutrition and Food Hygiene, School of Public Health, Harbin Medical University, 157 Baojian Road, Nangang District, Harbin, 150081, P. R. China; 2 Department of Epidemiology, School of Public Health, Harbin Medical University, 157 Baojian Road, Nangang District, Harbin, 150081, P. R. China; 3 Department of Public Health Surveillance, Harbin Center for Disease Control and Prevention, 30 Weixing Road, Daowai District, 150056, Harbin, P. R. China; 4 Department of Epidemiology and Biostatistics, School of Public Health, Harbin Medical University, 157 Baojian Road, Nangang District, Harbin, 150081, P. R. China

**Keywords:** biomarkers, calcium deficiency, metabonomics, UPLC/Q-TOF MS/MS, urine

## Abstract

**Background:**

Calcium deficiency is a global public-health problem. Although the initial stage of calcium deficiency can lead to metabolic alterations or potential pathological changes, calcium deficiency is difficult to diagnose accurately. Moreover, the details of the molecular mechanism of calcium deficiency remain somewhat elusive. To accurately assess and provide appropriate nutritional intervention, we carried out a global analysis of metabolic alterations in response to calcium deficiency.

**Methods:**

The metabolic alterations associated with calcium deficiency were first investigated in a rat model, using urinary metabonomics based on ultra-performance liquid chromatography coupled with quadrupole time-of-flight tandem mass spectrometry and multivariate statistical analysis. Correlations between dietary calcium intake and the biomarkers identified from the rat model were further analyzed to confirm the potential application of these biomarkers in humans.

**Results:**

Urinary metabolic-profiling analysis could preliminarily distinguish between calcium-deficient and non-deficient rats after a 2-week low-calcium diet. We established an integrated metabonomics strategy for identifying reliable biomarkers of calcium deficiency using a time-course analysis of discriminating metabolites in a low-calcium diet experiment, repeating the low-calcium diet experiment and performing a calcium-supplement experiment. In total, 27 biomarkers were identified, including glycine, oxoglutaric acid, pyrophosphoric acid, sebacic acid, pseudouridine, indoxyl sulfate, taurine, and phenylacetylglycine. The integrated urinary metabonomics analysis, which combined biomarkers with regular trends of change (types A, B, and C), could accurately assess calcium-deficient rats at different stages and clarify the dynamic pathophysiological changes and molecular mechanism of calcium deficiency in detail. Significant correlations between calcium intake and two biomarkers, pseudouridine (Pearson correlation, *r *= 0.53, *P *= 0.0001) and citrate (Pearson correlation, *r *= -0.43, *P *= 0.001), were further confirmed in 70 women.

**Conclusions:**

To our knowledge, this is the first report of reliable biomarkers of calcium deficiency, which were identified using an integrated strategy. The identified biomarkers give new insights into the pathophysiological changes and molecular mechanisms of calcium deficiency. The correlations between calcium intake and two of the biomarkers provide a rationale or potential for further assessment and elucidation of the metabolic responses of calcium deficiency in humans.

## Background

Calcium deficiency is a global public-health problem, especially in developing countries [1,2]. In China, the Nutrition and Health Status Survey in 2002 indicated that less than 10% of Chinese citizens had an adequate dietary intake of calcium; the average intake was 391 mg/day, which is only 41% of the recommended intake [3-5]. Chronic untreated calcium deficiency can cause many severe consequences, including osteomalacia (osteopenia) [6], rickets [7-9], and osteoporosis [2,10,11], and has received worldwide attention [2,5]. However, the diagnosis of calcium deficiency, particularly in the initial stages, is easily missed because this state is asymptomatic and does not lead to obvious pathological changes. However, even in the initial stage, metabolic alterations or potential dysfunctions have already occurred.

A number of methods are currently used to assess calcium nutrition status, including epidemiological survey, calcium-balance study [12-14], serum biochemical analysis [15,16], and radiological examination [17]; however, none of these is suitable for large-scale screening of calcium deficiency in a population. A calcium metabolic-balance study based on the dual-trace stable isotope technique is the classic method, and is considered the most effective method of assessing calcium nutrition status; however, this method is labor-intensive, lengthy, and costly [18]. Serum biochemistry testing is an unpleasant and invasive examination, especially for children, and its sensitivity and specificity are not always satisfactory [15,16]. Radiological changes or bone changes reflect only severe calcium deficiency, at which point there has already been serious damage to the body. Therefore, radiological examination, in general, cannot provide a diagnosis of calcium deficiency during the early stages, unless there is pathological injury to bone [17]. Moreover, a detailed understanding of the molecular mechanism of calcium deficiency has remained somewhat elusive. For the aforementioned reasons, a sensitive and non-invasive tool is needed to assess calcium nutrition status accurately and to identify biomarkers that can elucidate the mechanisms underlying calcium deficiency.

Metabonomics, which involves 'the quantitative measurement of the global, dynamic multi-parametric metabolic response of living systems to pathophysiological stimuli or perturbations of whatever source' [19] is an emerging science. Metabonomics has shown considerable potential in many applications, including identification of biomarkers, diagnosis, and prediction of disease, and elucidation of the dynamic responses of organisms to disease or environmental changes [20-26]. Owing to its high chromatographic resolution, high sensitivity, and rapid separation, ultra-performance liquid chromatography (UPLC) coupled with mass spectrometry (MS) has been used widely in metabonomics to investigate subtle metabolite alterations in complex mixtures [27-30]. Complex three-dimensional datasets (for example, retention time, mass:charge ratio, and intensity) with thousands of detectable ions can be obtained when biological specimens are analyzed using UPLC-MS. To reduce the complexity of MS spectra, simplify the data interpretation, and screen the potential biomarkers, investigators have used multivariate statistical methods such as principal component analysis (PCA) and partial least-squares discriminant analysis (PLS-DA) for metabonomics studies [31].

As a rule, urine is the best biological fluid to use for studying nutrient intake or identifying biomarkers. Urine is essentially the body's liquid waste repository, and any endogenous or exogenous metabolites that are not needed or are present in excess may be found in the urine [32]. Therefore, more compounds can be found in urine than in any other biological matrix. In addition, compared with other biological specimens, urine collection is non-invasive and is not volume-limited. Further, urine can easily be sampled in a serial manner, which allows the temporal metabolic changes to be studied [33]. Thus, urine is a key biological matrix in metabolic-profiling studies, and is considered a potential source of biomarkers. Urinary metabonomics have proven to be of great value in studies of drug toxicology [20,34], nutrition metabolism [35,36], and diagnosis of several diseases, including colorectal cancer [28,37], bladder cancer [38], and prostate cancer [39].

In the current study, we carried out a urinary metabonomics study on calcium-deficient rats, using UPLC and quadrupole time-of-flight tandem mass spectrometry (Q-TOF MS/MS) coupled with multivariate statistics to identify reliable biomarkers and to explore the metabolic alterations associated with the initiation and progression of calcium deficiency. We verified the reproducibility and reliability of the calcium-deficient model and biomarkers using a repeated low-calcium study, and we investigated the effect of calcium supplementation on the urinary metabolic profiling of calcium deficiency. We elucidated the implicated biological mechanisms and the biochemical pathways of the identified biomarkers, and analyzed the possible molecular mechanisms underlying the calcium deficiency. Furthermore, correlations between dietary calcium intakes and the identified biomarkers from rat models were investigated for their potential application to humans.

## Methods

### Ethics approval

The study was approved by the Harbin Medical University Institutional Animal Care Committee and performed in accordance with the Harbin Medical University guidelines for the care and use of laboratory animals.

### Chemicals and reagents

The products used in this study were high-performance liquid chromatography (HPLC)-grade acetonitrile and methanol (Honeywell Burdick & Jackson, Muskegon, MI, USA), HPLC-grade formic acid (Beijing Reagent Company, Beijing, China), and leucine-enkephalin (Sigma-Aldrich, St Louis, MO, USA). Ultrapure water was prepared in our laboratory (Ultra Clear System; Siemens Water Technologies, Nuremberg, Germany). Biomarker standards of the highest grade available were obtained from commercial sources.

### Animal experiments

Male Wistar rats aged 4 weeks (70 ± 10 g body weight; Shanghai Laboratory Animal Co. Ltd., Shanghai, China) were housed in individual metabolism cages at 21 ± 2°C and 50 ± 5% relative humidity with free access to food and distilled water for an adaptation period of 7 days. Food intake and body weight were recorded weekly.

#### Experiment I

The rats were divided randomly into two groups and fed on either a diet with either normal (AIN-93G Growth Purified Diet containing 0.50% (w/w) calcium) or low (AIN-93G Growth Purified Diet containing 0.15% (w/w) calcium) levels of calcium for 12 weeks (see Additional file 1). The low-calcium group (LCG) was the calcium-deficiency model (n = 24) and the normal-calcium group (NCG was the control (n = 24). Once a week, 24-h urine samples were collected in the metabolism cages for the metabonomics study. All urine samples were separated by centrifugation at 3,000 rpm (835 g) for 10 minutes, and the supernatant was stored at -80°C. At weeks 2, 4, 6, 8, 10 and 12, 24-h urine and feces samples were taken over 3 consecutive days from eight rats for the calcium metabolic-balance study. After the volumes of urine and the weights of feces were measured, they were stored at -80°C until measurement of calcium. At the end of weeks 4, 8, and 12, eight rats were selected randomly from each group, fasted for 12 hours, anesthetized by intraperitoneal injection of sodium pentobarbital (40 mg/kg body weight), and then killed by bloodletting. Blood samples were taken from the abdominal aorta, and immediately separated by centrifugation at 3,000 rpm (835 g) for 15 minutes. The sera were prepared for biochemical analysis. The left femur of each rat was isolated by dissection, and freed from the muscle and connective tissue.

##### Serum and urine analysis

Levels of serum calcium, phosphorus and alkaline phosphatase (AP) were measured with commercial kits (Nanjing Jiancheng Bioengineering Institute, Nanjing, China) using an automatic biochemical analyzer (AUTOLAB PM 4000; AMS Corporation, Rome, Italy). ELISA was used to assay serum parathyroid hormone (PTH; Alpco 31-IPTMS-E01 Mouse/Rat Intact PTH EIA (96 wells); ALPCO Diagnostics, Salem, NH, USA; inter-assay/intra-assay percentage coefficient of variation (% CV) < 6% and < 8%, respectively; detection limit 1.0 pg/ml), rat fibroblast growth factor 23 (FGF23, catalog number E90746Ra; % CV < 5% and< 8%, respectively; detection limit 6.1 pg/ml) and 1,25(OH)_2_D_3 _(catalog number: E90467Ge; %CV < 5% and < 7%, respectively; detection limit 4.1 pg/ml) (both Uscn Life Science Inc., Wuhan, China).

##### Calcium-balance study determination

The detailed experiment procedure has been described previously [13]. The calcium contents of urine, feces, and diet were measured with an atomic absorption spectrophotometer (AA-7010; East & West Analytical Instruments, Inc. Beijing, China).

##### Bone-mineral density measurement

The bone-mineral density (BMD) of the rat femurs was measured using dual energy X-ray absorptiometry (Norland XR-36 DEXA System; Cooper Surgical, Trumball, CT, USA) in the Second Affiliated Hospital of Harbin Medical University (Nangang District, China).

#### Experiment II

Experiment II was designed to verify the reproducibility and reliability of the calcium-deficient model and the identified biomarkers from experiment I. Thirty-six rats were divided randomly into two groups, NCG (*n *= 12) and LCG (*n *= 24), as before, and the diets described for experiment I were supplied to the two groups for 8 weeks. At the end of week 8, the LCG rats were divided randomly into two sub-groups: the maintained low-calcium group (MLCG; *n *= 12) and the calcium-supplementation group (CSG; *n *= 12). The CSG rats were fed a different diet, with a calcium content of 1.5% (w/w) (see Additional file 1), while the diets supplied for the NCG and MLCG rats were unchanged. All rats were fed for another 4 weeks. The 24-h urine samples were collected as described for experiment I during weeks 1-12.

### Global metabolic-profiling analysis of rat urine by UPLC/Q-TOF MS/MS

#### Sample preparation

Urine samples were allowed to thaw at room temperature, diluted 1:1 (v/v) with water, mixed by vortex for 1 minute, and then separated by centrifugation at 12,000 rpm (13362 g) for 10 minutes. The supernatants were then transferred to autosampler vials.

#### UPLC conditions

Chromatographic separation was performed on a 1.8 μm T_3 _column (ACQUITY (HSS); Waters Corp., Milford, MA, USA; 2.1 mm × 100 mm) equipped with a UPLC system (ACQUITY UPLC; Waters Corp., USA). The temperatures of the column and autosampler were maintained at 35°C and 4°C, respectively. A sample (2 μl) of the urine supernatant obtained by centrifugation was injected onto the column at a flow rate of 0.35 ml/min. The mobile phase consisted of water containing 0.1% formic acid (solution A) and acetonitrile (solution B). The elution gradient was as follows: 2% B for 0.5 minutes; 2 to 20% B over 0.5 to 6.0 minutes; 20 to 35% B over 6.0 to 7.0 minutes; 35 to 70% B over 7.0 to 9.0 minutes; 70 to 98% B over 9.0 to 10.5 minutes; 98% B for 2.0 minutes;, then return to 2% B for 6.0 minutes. Once the initial settings were established, the column was equilibrated for 2.0 minutes. Acetonitrile was run every fifth sample as a blank solution, and the urine samples in the two analysis batches (experiment I and II) were injected alternately as five NCG and five LCG samples.

#### Mass spectrometry conditions

Q-TOF MS/MS was performed with a mass spectrometer (Micromass Q-TOF mass spectrometer; Waters Corp., Manchester, UK) using an electrospray ionization (ESI) interface with the ESI source operated in the negative ion mode (ESI^-^). The analytical parameters were as follows: capillary voltage, 2800 V; sample cone voltage, 35 V; collision energy, 6 eV; source temperature, 100°C; desolvation gas (nitrogen) flow, 650 L/h; desolvation temperature, 320°C; cone gas (nitrogen) flow, 50 l/h; collision gas, argon; MCP detector voltage, 2200 V. The Q-TOF mass acquisition rate was set at 0.4 seconds with an interscan delay of 0.1 second. The scan mass range was from 50 to 1000 m/z. The Q-TOF MS/MS data were collected in centroid mode, using the lock spray to ensure accuracy and reproducibility. A concentration of 200 pg/ml leucine-enkephalin was used as lock mass (m/z 554.2615) in ESI^-^. The lock spray frequency was set at 10 seconds, and the lock mass data were averaged over 10 scans for correction. The MS/MS spectra of potential biomarkers were obtained by UPLC-MS/MS.

#### Data processing

The raw data were imported into MarkerLynx software (which is incorporated in the MassLynx software; version 4.1; SCN 714; Waters Corp., USA). The MarkerLynx ApexTrack peak integration was used for peak detection and alignment. The ApexTrack peak parameters were set as follows: peak width at 5% height, 1 second, and peak-to-peak baseline noise (calculated automatically). Collection parameters were set as follows: mass window, 0.05 Da; retention time window, 0.2 minutes; minimum intensity, 80; noise elimination level, 6.0; deisotope data, Yes. Data were only used for the period 0.4 to 10.5 minutes; that is, up to the point at which the column-washing phase of the analysis started. After being recognized and aligned, the intensity of each ion was normalized to the summed total ion intensity of each chromatogram, to take into account the variation in urine concentration and volume. The data-reduction process was handled in accordance with the '80% rule' [40]. The three-dimensional data including peak number (RT-m/z pair), sample name, and normalized peak areas were exported to the EZinfo software (version 2.0.0.0; June 5 2008; Waters Corp., USA) for multivariate statistical analysis. The data were mean-centered and Pareto-scaled before multivariate statistical analysis. PCA, an unsupervised multivariate statistical approach, can reduce the dimensionalities of complex datasets and provide an overview of all observations in data tables, such as groupings, trends, and outliers of the different groups of samples [30,31]. The PLS method was used for the analysis of time changes [24]. After an initial overview of the UPLC/Q-TOF-MS data, the supervised partial least-squares discriminant analysis (PLS-DA) and orthogonal projection to latent structures discriminant analysis (OPLS-DA) were applied to visualize the maximal difference associated with calcium deficiency for each LCG versus NCG at all time points. A default seven-fold (Leave-1/7th Samples-Out) cross-validation procedure and testing with 100 random permutations were performed to avoid the over-fitting of supervised PLS-DA models, using SIMCA-P software (version 11.5; Umetrics AB, Umeå, Sweden).

#### Identification of biomarkers and metabolic pathway analysis

The probable empirical formulas of the biomarkers were first derived based on accurate mass measurement (mass error of <20 ppm) and by considering the relative intensities of the isotope peaks through the high-resolution MS spectra. The MassFragment™ application manager (MassLynx v4.1, Waters Corp., USA) was used to facilitate the MS/MS fragment ion-analysis process by way of chemically intelligent peak-matching algorithms. The identification of potential biomarkers was achieved by comparison with free online databases, such as ChemSpider (http://www.chemspider.com), the Human Metabolome Database (HMDB) (http://www.hmdb.ca), Metlin (http://metlin.scripps.edu/) and Pubchem (http://pubchem.ncbi.nlm.nih.gov/), using exact mass and MS/MS spectra. Finally, the biomarkers were further confirmed by standard compounds based on both retention times and MS/MS spectra.

The implicated pathways of biomarkers were interpreted using databases, including HMDB and KEGG.

### Human study

Because the ultimate goal of this non-invasive technique is to screen for calcium deficiency in humans, we further confirmed the potential application of three of the biomarkers identified from the rat models in calcium-deficient human subjects. Post-menopausal women have been reported to be the core of the population at risk for calcium deficiency, thus, to investigate the correlations between calcium intake and the identified biomarkers of calcium deficiency, we chose post-menopausal women as the subjects in this study.

The project was approved by the Ethics Committee of Harbin Medical University, and informed consent was obtained from all subjects before entry into the study.

In total, 70 participants who had lived in Harbin for more than 5 years were randomly recruited (from Hongqi Community Health Service Center, Harbin City, China). All the participants were women aged 50 to 64 years (mean ± SD 56.55 ± 3.8 years), had natural menopause for up to 1 year or were post-menopausal.

The exclusion criteria included subjects with: any conditions affecting calcium absorption or metabolism, such as hyperparathyroidism, inherited bone disease, ovarian surgery, gastrointestinal resection, thyroid resection, malabsorption, or diseases of the digestive tract; (2) other diseases, such as coronary heart disease, stroke, diabetes, cancer, thyroid or parathyroid disease, and chronic disease of the liver or kidney; or current treatment with hormone-based drugs or other medications such as estrogen.

The average calcium intake per day was derived from 3 days of weighed dietary records for each woman, and 24-h urine samples were collected from all subjects. The normalized peak areas of the biomarkers were detected for quantitative analysis by UPLC/Q-TOF MS/MS.

### Statistical analysis

Serum biochemical indicators and BMDs of the LCG and NCG rats were expressed as the mean ± SD. Differences between the two groups were analyzed by the independent *t*-test and Mann-Whitney *U*-test. Correlations between dietary calcium intake and the biomarkers in human subjects were examined by linear regression analysis. Log-transformed values were used for the skewed distributions of the biomarkers. A two-tailed value of *P <*0.05 was considered significant. All analyses including the error bars of three unique metabolites were performed using SPSS software (version 16.0; SPSS Inc., Chicago, IL, USA).

## Results

### Serum biochemical indicators and bone-mineral density

There was a significant difference between the LCG and NCG rats for mean serum PTH and 1,25(OH)_2_D_3 _at week 4, for serum AP, PTH, and 1,25(OH)_2_D_3 _at week 8, and for all of the serum indicators plus BMD at week 12 (Table 1). The serum level of FGF23 at week 12 was significantly higher in the LCG rats compared with the NCG rats (*P *= 0.002). There was no significant difference between the two groups in dietary intake or body weight at any time point. Calcium excretion in urine and feces, and calcium intake were significantly lower in the LCG rats compared with the NCG rats at weeks 2, 4, 6, 8, 10, and 12 (*P*<0.05) (see Additional file 2). Although the apparent absorptivity of calcium was significantly higher in the LCG rats compared with the NCG rats at weeks 2, 4, 6, 8, 10, and 12, the retention (absorption) of calcium was significantly lower in the LCG rats (*P*<0.05). These results confirmed the successful establishment of the calcium-deficient rat model in our study.

**Table 1:** Serum biological indicators and bone-mineral density between rats on the normal-calcium diet and rats on thelow-calcium diet.

	Normal-calcium group			Low-calcium group		
		
	Week 4	Week 8	Week12	Week 4	Week 8	Week 12
Calcium, mmol/l	2.53 ± 0.26	2.55 ± 0.19	2.56 ± 0.28	2.55 ± 0.23	2.51 ± 0.17	2.31 ± 0.28*
Phosphorus, mg/l	9.38 ± 1.94	9.29 ± 2.35	9.17 ± 2.11	9.29 ± 2.03	8.85 ± 1.60	8.67 ± 2.03
AP, U/l	85.8 ± 21.0	82.4 ± 14.2	80.1 ± 16.3	96.6 ± 17.9	111.7 ± 20.3*	117.8 ± 17.9*
PTH, pg/ml	45.6 ± 6.08	42.3 ± 2.22	31.2 ± 1.75	59.4 ± 6.87*	53.7 ± 1.95*	42.8 ± 2.13*
1,25, OH_2_D_3_, pg/ml	70.7 ± 14.9	71.6 ± 18.1	72.2 ± 19.9	87.2 ± 23.1*	120.2 ± 26.4*	121.3 ± 19.1*
FGF23, pg/ml	-	-	211 ± 9.28	-	-	234 ± 14.44*
BMD, g/cm^2^	0.258 ± 0.02	0.285 ± 0.02	0.298 ± 0.02	0.254 ± 0.03	0.277 ± 0.01	0.262 ± 0.03*

Abbreviations: AP, alkaline phosphatase; BMD, bone-mineral density; FGF23, fibroblast growth factor 23; LCG, low-calcium group; NCG, normal-calcium group, PTH, parathyroid hormone. *Significant differences (*P*<0.05) for mean values of LCG compared with NCG at the same time points.

### Quality assessment of the metabonomics platform

To verify the reproducibility and reliability of the data, a representative pooled quality control (QC) sample was prepared by mixing equal volumes of the urine samples from five LCG rats and five NCG rats; this was analyzed as every 15th sample throughout the analytical run.

An initial overview of the quality of the analytical run was obtained by PCA of the sample dataset that included the QC injections. The QC results were clustered tightly in the middle of the scores plot (see Additional file 3; red stars). The relative standard deviations (RSD; %) of the retention time and peak area in intra-batch/inter-batch assays ranged from 0 to 0.73 and 0.8 to 4, respectively, in the intra-batch assay, and from 0.1 to 2.7 and 1.5 to 6.2, respectively in the inter-batch assay (see Additional file 3). The results showed that the stability of the metabonomics platform was excellent throughout the run, and was sufficient to ensure the data quality for further global metabonomics analysis.

### Urinary metabolic-profiling analysis of experiment I

**Figure 1:**
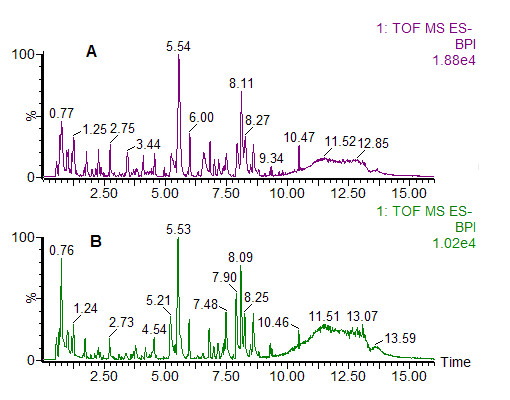
**Representative ultra-performance liquid chromatography and quadrupole time-of-flight tandem mass spectrometry (UPLC/Q-TOF MS/MS)-based peak intensity chromatogram of rat urine in electrospray ionization (ESI) negative ion mode**. **(A) **Rat on normal-calcium diet; **(B) **rat on low-calcium diet. BPI, baseline peak intensity.

All of the urine samples (n = 384) from weeks 1-12 collected in experiment I were analyzed by UPLC/Q-TOF MS/MS in ESI^-^. Representative chromatograms of baseline peak intensity (BPI) of urine indicated that the metabolites of the samples attained suitable separation on the UPLC T_3 _column by gradient elution (Figure 1). After the data reduction was performed in accordance with the '80% rule', 3131 variables were used for multivariate statistical analysis.

**Figure 2:**
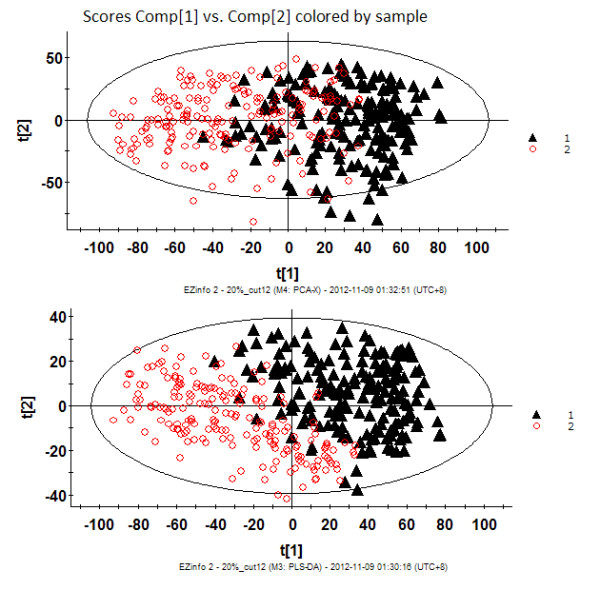
**Urinary metabolic-profiling analysis of rats in the low-calcium group (LCG) and normal-calcium group (NCG) during weeks 1 to 12**. **(A) **Principal component analysis (PCA) score plots; **(B) **partial least-squares discriminant analysis (PLS-DA) score plots. LCG rats are indicated by black triangles and NCG rats by red circles. Each data point represents one subject. Comp, component. t[1], component 1; t[2], component 2.

To visualize the general clustering trends between the two groups, PCA and PLS-DA were applied to the urinary metabolic profiling obtained from the NCG and LCG rats. The PCA scores plot (the R2Y value represents the goodness of fit of the model, and the Q2 value represents the predictability of the models) for the first two components: R2Y = 0.280, Q2 = 0.236) reflected a separation trend between the LCG and NCG rats (Figure 2A). As shown by the PLS-DA scores plot (for the first five components, R2Y = 0.826 and Q2 = 0.687; Figure 2B), the NCG and LCG rats could be separated into distinct clusters, with distinct metabolic alterations between the two groups. To assess the risk that the current PLS-DA model was spurious, the permutation test for PLS-DA was applied. All R2X and Q2 values to the left were lower than the original points to the right (see Additional file 4), showing that the PLS-DA model was valid. The results suggested that the low-calcium diet had led to the urinary metabolite alterations in our rat model.

**Figure 3:**
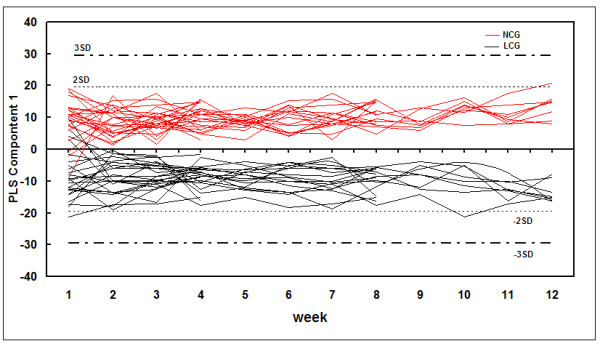
**Batch partial least-squares (PLS) scores plots of urine samples mapped with time**. Dashed horizontal lines show two and three standard deviations (SDs) for the dataset. Low-calcium group (LCG) rats are indicated by black lines and normal-calcium group (NCG) rats by red lines.

**Figure 4:**
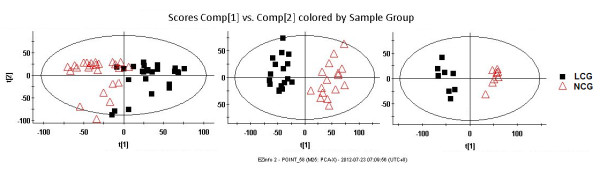
**Principal component analysis (PCA) score plots of rats in the low-calcium group (LCG) and normal-calcium group (NCG)**. **(A-C) **Week 2, n = 48 (for two components, R2Y = 45.0%, Q2 = 24.1%); week 4, n = 32 (for three components R2Y = 54.6%, Q2 = 27.6%) and week 9, n = 16 (for three components R2Y = 58.3%, Q2 = 32.1%). The R2Y value represents the goodness of fit of the model, and the Q2 value represents the predictability of the models. LCG rats are indicated by black squares and NCG rats by red triangles. Each data point represents one subject. Comp, component. t[1], component 1; t[2], component 2.

To visualize the trend changes between the two groups over time, batch PLS analysis was applied to the urinary metabolic profiling obtained from the LCG and NCG rats. Excluding the first week, the batch PLS scores plot (Figure 3) showed a clear separation tendency between the two groups from weeks 2 to 12. To investigate the global metabolic alterations between the two groups, the UPLC/Q-TOF MS/MS data for all time points were analyzed by PCA. A small separation tendency between the two groups was seen at week 2 (Figure 4A), with clear separation between the two groups at week 4 (Figure 4B). The metabolic profiling had changed more obviously at week 9 (Figure 4C), which led to further separation between the two groups. Based on these results, the urinary metabolite profiling of the LCG rats was altered by the second week of the low-calcium diet. These results provide evidence of the important potential value of urinary metabolic profiling for the diagnosis of calcium deficiency.

**Figure 5:**
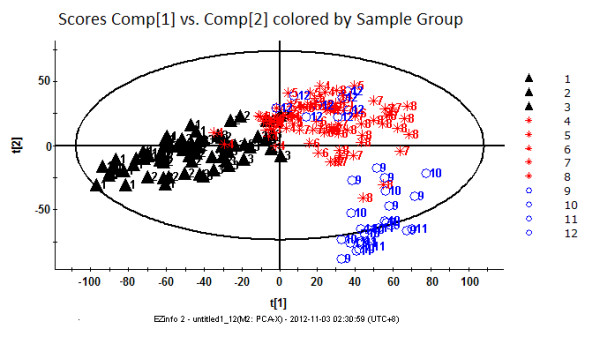
**Metabolic trajectory scores plots by principal component analysis (PCA) model derived from the urine of rats on low-calcium diet**. Samples were collected during weeks 1 to 12. Black triangle: weeks 1 to 3; red star: weeks 4 to 8; blue circle: weeks 9 to 12. Comp, component. t[1], component 1; t[2], component 2.

To investigate the dynamic and continuous alteration in urinary metabolites during the initiation and progression of calcium deficiency, a metabolic trajectory analysis was conducted using PCA for the global urinary metabolic-profiling analysis of the LCG rats during weeks 1 to 12. The observations at weeks 4 to 8 and 9 to 12 markedly deviated from those at weeks 1 to 3 (Figure 5). There were three clear clusters for the plots of the metabolic trajectory scores for weeks 1 to 3, 4 to 8, and 9 to 12, which implied that there were three different developmental stages for the progression of calcium deficiency, that is, an early stage (weeks 1 to 3), a middle stage (weeks 4 to 8) and a late stage (weeks 9 to 12). These results showed that a continuous low-calcium diet could induce persistent underlying metabolic variations and worsening of calcium deficiency.

To identify the difference in metabolites between the NCG and LCG rats, OPLS-DA models were constructed for all time points. Discriminating variables were selected primarily according to their variable importance in projection values (VIP > 1.5) and S-plot by OPLS-DA. The discriminating variables obtained from the OPLS-DA models were further validated by the Wilcoxon test (*P*<0.05). Although hundreds of discriminating variables were selected as potential markers at each time point, only a small percentage were shared across different time points. It was expected that the real biomarkers should change in accordance with the progression of calcium deficiency, therefore, only the biomarkers with regular trends of change were considered as potential biomarkers in this study.

**Figure 6:**
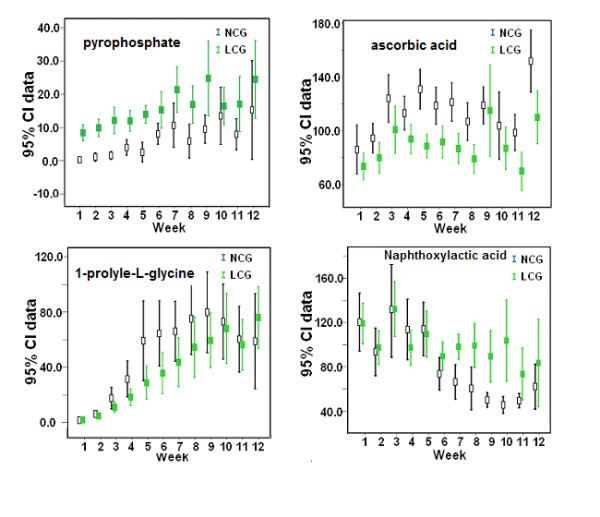
**Error bar plots for the peak areas of four potential biomarkers with representative changing trends**. The normalized peak area is shown on the vertical coordinate, and the time points of sample collection on the horizontal coordinate. Error bars represent the means and 95% confidence interval (CI) of the normalized peak areas. Low-calcium group (LCG) rats are indicated by green boxes and normal-calcium group (NCG) rats by black boxes.

Three distinct changing trends for potential biomarkers were obtained by time-course analysis. As shown in Figure 6A1 and 6A2, Biomarkers whose peak areas were consistently higher or lower in the LCG rats compared with the NCG rats throughout the experiment (see Figure 6A1, 6A2)., were defined as type A. Biomarkers whose peak areas were lower in the LCG rats than in the NCG rats for weeks 2 to 9, and subsequently disappeared in weeks 10-12, (that is, the he changing trend for the biomarker was that it appeared at the initial stage and disappeared at the middle or late stage) were defined as type B (Figure 6B). Finally, biomarkers whose peak area was higher in the LCG rats than in the NCG rats from week 6 to the end of the experiment were defined as type C (Figure 6C). Based on these three changing trends, 38 potential biomarkers associated with calcium deficiency were selected (see Additional file 5).

### Urinary metabolic-profiling analysis of experiment II (repeated low-calcium diet experiment)

The analytical method of urinary metabolite profiling used in experiment I was used in the repeated low-calcium diet experiment (urine samples, n = 288). Similar results were obtained for classification trends by PCA, PLS-DA, batch PLS scores plot, and PCA trajectory analysis (see Additional file 6), which indicated that the urinary metabolic profiling of calcium-deficient rats had a high degree of repeatability. These similar results further confirmed that the cluster between the two groups was robust and valid, and that the urinary metabolite profiling of calcium-deficient rats was significantly altered by the second week of the low-calcium diet. Most of the biomarkers from experiment I were identified, and these had the same changing trends as seen in the earlier experiment. Only nine biomarkers were not seen in the repeated low-calcium diet experiment, and these were excluded. Accordingly, the list of markers now comprised 29 potential biomarkers (see Additional file 7).

### Urinary metabolic-profiling analysis of experiment II (calcium-supplement experiment)

Significant changes in several serum biological indicators and a decreasing trend for BMD were found in the LCG rats after week 8 (Table 1). Moreover, based on the metabolic trajectory analysis by PCA (Figure 5), it seemed that calcium deficiency would progress from the middle stage to the late stage if the LCG rats were kept on a low-calcium diet after week 8. Therefore, to investigate the effect of calcium supplementation on the urinary metabolic profiling of calcium-deficient rats, half of the LCG rats (CSG) were supplied with the 1.5% high-calcium diet after week 8, while the other half (MLCG) remained on the low-calcium diet.

**Figure 7:**
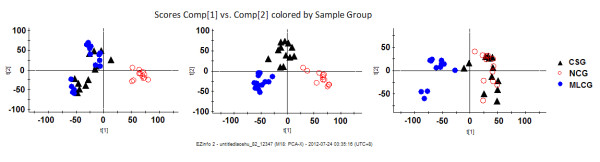
**Principal component analysis (PCA) score plots of low-calcium diet rats, normal-calcium diet rats and calcium-supplement diet rats (n = 36)**. The maintained low-calcium group (MLCG) rats are indicated by blue circles, the normal-calcium group (NCG) rats by red circles, and the calcium-supplement group (CSG) by black triangles. The R2Y value represents the goodness of fit of the model, and the Q2 value represents the predictability of the models. **(A) **Before calcium supplementation (for six components R2Y = 53.9%, Q2 = 23.4%); **(B) **after calcium supplementation for 1 week (for five components R2Y = 55.3%, Q2 = 26.1%); **(C) **after calcium supplementation for 4 weeks (for five components R2Y = 55.6%, Q2 = 28.3%). Comp, component. t[1], component 1; t[2], component 2.

The PCA score plots showed that the projections for the metabolites measured in CSG rats clustered with those of the LCG rats and deviated from those of the NCG rats before calcium supplementation at week 8 (Figure 7A). After calcium supplementation for 1 week, three clusters were apparent in the urinary metabolic profiling, with the CSG rats clearly discriminated from both the MLCG and NCG rats (Figure 7B). This result indicated that the calcium deficiency had been alleviated by the calcium supplementation, but that the calcium metabolism had not yet returned to normal levels. After calcium supplementation for 4 weeks, the CSG rats were completely differentiated from the MLCG rats, and were clustered with the NCG rats (Figure 7C), indicating that the calcium metabolic alterations had been largely restored, and that calcium metabolism was nearly normal.

Accompanying the visual changes in urinary metabolic profiling, the potential biomarkers also changed significantly in the CSG rats compared with tthe MLCG rats. For example, the peak area of the biomarker with m/z 74.0238 in the CSG rats decreased significantly at the end of week 1 and disappeared at the end of week 2. This was the type of calcium-deficiency biomarker that we were seeking. If there were still significant differences in the peak areas of biomarkers between the CSG and NCG rats at week 4 of calcium supplementation, these biomarkers were excluded from the biomarker list. Based on this, 2 more potential biomarkers were excluded, leaving 27 potential calcium-deficiency biomarkers (Table 2). When these were assessed, it was found that, compared with the NCG rats, 12 biomarkers were upregulated and 15 biomarkers were downregulated in the LCG rats.

**Table 2:** Identifications and changing trends for the biomarkers of calcium deficiency.

**No**.	RT (min)	Actual mass(Da)	Exact mass(Da)	Mass error, ppm	Molecule composition	Identity	Trend^b^	Onset	Disappear	Type
1	1.21	191.0173	191.0192	9.9	C_6_H_8_O_7_	Citrate^b^	↓	1	12	A
2	1.20	111.0073	111.0082	8.1	C_5_H_4_O_3_	Ion fragment of citrate^b^	↓	1	12	A
3	0.68	176.9366	176.9354	6.8	H_4_P_2_O_7_	Pyrophosphoric acid^b^	↑	1	12	A
4	0.98	145.0158	145.0137	14.5	C_5_H_6_O_5_	Oxoglutaric acid^c^	↓	1	12	A
5	0.96	175.0251	175.0243	4.5	C_6_H_8_O_6_	Ascorbic acid^b^	↓	1	12	A
6	5.89	192.0629	192.0661	16.7	C_10_H_11_NO_3_	Phenylacetylglycine^c^	↑	1	12	A
7	5.97	74.0238	74.0242	5.4	C_2_H_5_NO_2_	Glycine^b^	↑	1	12	A
8	1.02	101.0237	101.0239	2.0	C_4_H_6_O_3_	Acetoacetic acid^c^	↓	1	12	A
9	6.42	283.0714	283.0679	12.4	C_10_H_12_N_4_O_6_	Xanthosine^c^	↑	1	12	A
10	6.81	231.0702	231.0657	19.5	C_13_H_12_O_4_	Naphthoxylactic acid^d^	↑	6	12	C
11	9.30	407.2809	407.2797	2.9	C_24_H_40_O_5_	Cholanoic acid**^c^**	↓	5	12	C
12	1.14	167.0211	167.0205	3.6	C_5_H_4_N_4_O_3_	Uric acid^b^	↓	1	5	B
13	0.99	293.0520	293.0484	12.3	C_9_H_14_N_2_O_7_S	L-Glutamic acid, N-[[[(1S)-1-carboxy-2-mercapto-ethyl]amino]carbonyl]-**^d^**	↓	1	12	A
14	1.02	243.0625	243.0617	3.3	C_9_H_12_N_2_O_6_	Pseudouridine^b^	↓	8	12	C
15	8.19	201.1134	201.1127	3.5	C_10_H_18_O_4_	Sebacic acid^c^	↓	1	7	B
16	6.44	113.0231	113.0239	7.1	C_5_H_6_O_3_	4-Oxo-2-pentenoic acid**^d^**	↑	1	12	A
17	0.65	124.0059	124.0068	7.3	C_2_H_7_NO_3_S	Taurine^b^	↓	2	12	A
18	8.21	199.1352	199.1334	9.0	C_11_H_20_O_3_	6-(2-hydroxycyclopentyl) hexanoic acid**^d^**	↓	1	12	A
19	6.43	212.0020	212.0018	0.9	C_8_H_7_NO_4_S	Indoxyl sulfate^c^	↑	1	8	A
20	6.44	175.0236	175.0243	4.0	C_6_H_8_O_6_	D-glucurono-6,3-lactone^c^	↑	1	12	A
21	7.15	171.1008	171.1008	0	C_7_H_12_N_2_O_3_	Glycylproline^c^	↓	2	9	B
22	5.87	222.0808	222.0766	18.7	C_11_H_13_NO_4_	*N*-acetyl-L-tyrosine^c^	↓	2	9	B
23	8.46	113.0964	113.0966	1.8	C_7_H_14_O	Ethyl isobutyl ketone ^d^	↓	1	12	A
24	2.36	328.0451	328.0447	1.2	C_10_H_11_N_5_O_6_P	Cyclic adenosine monophosphate^b^	↑	1	12	A
25	4.31	178.0499	178.0504	2.8	C_9_H_10_NO_3_	Hippuric acid^b^	↑	1	12	A
26	1.08	129.0170	129.0188	14	C_5_H_6_O_4_	Monomethyl fumarate^d^	↑	6	12	C
27	0.67	194.9456	194.9460	2.1	H_6_P_2_O_8_	Phosphoric acid^b^	↑	1	12	A

Abbreviations: RT, retention time (minute). ^a^Arrow denoted the increase or decrease of the metabolites in low-calcium diet rat compared with those in the control rats. Type A: the biomarkers seen throughout the progression of calcium deficiency. Type B: the biomarkers appeared at the initial stage and dizappeared at the middle or late stage. Type C: the biomarkers seen at the middle or late stage. ^b ^Biomarkers confirmed using standard samples. ^c ^Biomarkers identified by tandem mass spectrometry (MS/MS) spectra of databases. ^d ^The possible elemental compositions of biomarkers determined based on exact mass data and MS fragmentation.

### Identification of biomarkers

In this study, 27 metabolites were selected as calcium deficiency biomarkers. Eleven biomarkers were confirmed by comparing their retention times and MS/MS fragmentation patterns with those of the standards (Table 2). Detailed identification experiments using Q-TOF MS/MS and standards to elucidate the elemental composition of each biomarker (for example, cAMP) were performed (see Additional file 8). Ten further biomarkers were identified by searching several free online databases. Hence, 21 biomarkers were identified (Table 2). Six more biomarkers were determined by exact mass, isotopic distribution, and mass spectra fragmentation patterns, using MassFragment software (for detailed data, see Additional file 9).

### Correlation between dietary calcium intake and each metabolite in human

**Figure 8:**
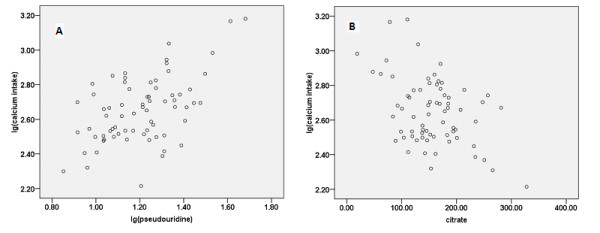
**Correlation between calcium intake and two biomarkers**. **(A, B) **The human study enrolled 70 women, and analzed the correclation between the log (lg) dietary calcium intakes and **(A) **urine pseudouridine (*r *= 0.53, *P *= 0.0001) or **(B) **citrate (*r *= -0.43, *P *= 0.001).

The average dietary calcium intake of the 70 women was 483 ± 248 mg (range 164 to 1517 mg). There were significant correlations between dietary calcium intake and urinary levels of citrate (Pearson correlation, *r *= -0.43, *P *= 0.001) and pseudouridine (Pearson correlation, *r *= 0.53, *P *= 0.0001) (Figure 8). However, no significant correlation between urinary pyrophosphoric acid (Pearson correlation, *r *= 0.15, *P *= 0.413) and dietary calcium intake was found (Figure 8).

## Discussion

Calcium deficiency is reversible when diagnosed at an early stage. However, without proper intervention, calcium deficiency can develop to an irreversible stage, such as rickets. Several methods, including epidemiological survey, calcium-balance study, serum biochemical investigations, and radiological examination, are currently used for the assessment of calcium nutrition status. However, none of these methods is suitable for large-scale population screening or for the clinical diagnosis of calcium deficiency at an early stage. Therefore, a non-invasive method and sufficiently sensitive and specific biomarkers are urgently needed for assessing the presence and progression of calcium deficiency. Urinary metabonomics based on UPLC/Q-TOF MS/MS has been of great value in the discovery of biomarkers and the elucidation of the pathogenesis of various diseases [37-39]. To date, there has been no report of urinary metabonomics focused on calcium deficiency. Therefore, this unbiased global urinary metabonomics study based on UPLC/Q-TOF MS/MS coupled with multivariate statistical analysis is the first to identify potential biomarkers and unravel the molecular mechanisms of calcium deficiency.

As shown by our experiments, there were no significant changes in the serum levels of phosphate, calcium, or AP before week 9, but there were changed in PTH and 1,25(OH)VD_3 _levels at this point. Although PTH secretion was exquisitely sensitive to very small changes in serum calcium concentration, PTH is not a specific biomarker of calcium deficiency. Numerous experiments have shown that a rise in PTH can be caused by other factors, such as primary hyperparathyroidism and secondary hyperparathyroidism [41,42]. Although the calcium balance changed significantly during the study, this method took at least 3 days to perform. In addition, the serum calcium level was significantly decreased until week 12, indicating the body's strong ability to maintain calcium homeostasis. Similarly, significant decreases in BMD were not found until week 12. These results suggest that both serum calcium and bone radiological change can only reflect long-term or severe calcium deficiency. In contrast to the serum indicators, urinary metabolic alterations between the NCG and LCG rats were detected at week 2 using global urinary metabolic profiling. This observation was also confirmed by a calcium-balance study. Moreover, after calcium supplementation for 1 week, the altered calcium metabolism had been largely restored. These results show that urinary metabolic profiling has high sensitivity to subtle metabolic perturbation. We were able to preliminarily distinguish the LCG from the NCG rats by urinary metabolic-profiling analysis.

To gain insight into the metabolic mechanism of calcium deficiency and to provide an accurate assessment of calcium nutrition status, reliable biomarkers should be identified. However, in metabonomics studies, a major challenge to date has been how to distinguish reliable biomarkers that are closely associated with the initiation and progress of disease from those that are altered but are unrelated to the disease. The ideal strategy for discovering reliable biomarkers should include a time-course analysis for discriminating metabolites in a experiment on rats with low-calcium diet, and subsequent validation of the candidate biomarkers by a repeated experiment on rats with low-calcium diet along with a nutrition-supplement experiment. Therefore, we developed an integrated screening method consisting of the following steps. First, the discriminating variables with regular and reasonable changing trends (types A, B and C; Figure 6), were selected as potential biomarkers by time-course analysis. Second, the repeatability of biomarkers is crucial for their identification and biological interpretation in metabonomics studies. Thus, to verify the repeatability of biomarkers, the same urinary metabolic profiling and time-course analysis of discriminating variables were applied in a repeated low-calcium experiment. Third, a calcium-supplement experiment based on the low-calcium experiment was used to confirm the above biomarkers.

As shown by our study results, 27 variables were chosen as reliable biomarkers of calcium deficiency. Biomarkers with the type A changing trend (for example, glycine, sebacic acid) were present throughout the progression of calcium deficiency. Biomarkers with the type B changing trend were defined as the specific biomarkers that were present in the early (for example, uric acid, weeks 1-5), early and middle (for example, indoxyl sulfate, weeks 1-8) stages of calcium deficiency. By contrast, biomarkers with the type C changing trend were defined as the specific biomarkers that were present in the middle (for example, cholanoic acid, weeks 5-12), middle and late (for example, pseudouridine, weeks 8-12) stages. Hence, we concluded that if significantly increased levels of glycine (type A) and decreased levels of uric acid (type B) were both detected, but biomarkers with the type C changing trend were not detected, this would suggest that the body was in the early stages of calcium deficiency. If significantly increased levels of glycine (type A) and indoxyl sulfate (type B), and decreased levels of ions (cholanoic acid; type C) were all detected in urine, this would indicate that the body was in the middle stage of calcium deficiency. Finally, if significantly increased levels of glycine (type A) and decreased levels of uridine (type C) were detected, this would suggest severe calcium deficiency. These results proved that the biomarkers identified by urinary metabolomics were sufficient to assess the level of calcium deficiency at different stages in the male rat, which cannot be achieved by serum biochemical analysis. After further validation of several biomarkers (oxoglutaric acid, phenylacetylglycine, pyrophosphoric acid, citric acid, sebacic acid, pseudouridine, indoxyl sulfate, and taurine) that occur frequently in humans, these biomarkers could be used alone or in combination as a non-invasive method with greater sensitivity and specificity for the diagnosis of calcium deficiency.

**Figure 9:**
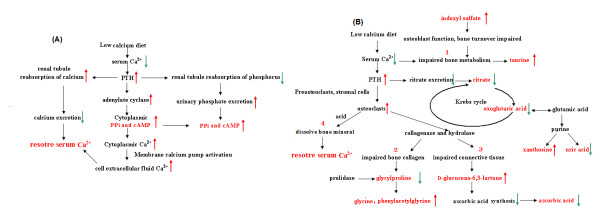
**Schematic of altered metabolic pathways associated with calcium deficiency based on urinary metabolites analyzed by ultra-performance liquid chromatography and quadrupole time-of-flight tandem mass spectrometry (UPLC/Q-TOF MS/MS)**. **(A) **Metabolism of calcium and phosphorus metabolism; **(B) **Krebs cycle and metabolism of bone collagen, purines and other biomarkers. Biomarkers are shown in red. Red arrows indicate increased levels and green arrows indicate decreased levels of these metabolites in the low-calcium group (LCG) rats compared with the control (normal-calcium group; NCG) rats. Black arrows indicate the reaction direction. The results of steps 1, 2, 3, and 4 steps lead to disorders of bone metabolism and decreased bone-mineral density.

Based on the biomarkers identified here (Figure 9), several metabolic pathways seem to be correlated with calcium metabolism. The increased levels of pyrophosphate, cAMP, and phosphate in the urine of the LCG rats suggest impaired calcium and phosphorus metabolism. The increased secretion of PTH in response to the low-calcium diet was responsible for the activation of adenylate cyclase and the production of cytoplasmic cAMP and pyrophosphate. The activation of this signal-transduction pathway would induce intracellular calcium mobilization and activation of the membrane calcium pump, which could result in an increased concentration of cytoplasmic Ca^2+ ^[43]. Cytoplasmic Ca^2+ ^was transported to the extracellular fluid of the cell in order to restore serum calcium levels when the rats were fed a low-calcium diet. By contrast, increased PTH and 1,25(OH)_2 _VD_3 _could promote renal tubule re-absorption of calcium and inhibit the re-absorption of phosphorus, thereby reducing urinary calcium excretion and increasing urinary phosphate excretion. Increased serum FGF23 led to decreased serum phosphorus and increased urine phosphorus levels [44]. Therefore, increased levels of inorganic phosphates, including phosphate, pyrophosphate, and cAMP, were found in the urine samples in this study (Figure 9A).

A high concentration of PTH can promote the transformation of preosteoclasts and stromal cells into osteoclasts. Highly active osteoclasts dissolve bone minerals by secreting acid onto the isolated surface, and simultaneously break down the bone matrix by secreting collagenase and hydrolase. Increased levels of glycylproline, the end product of collagen metabolism, were found when bone collagen was disrupted. Glycylproline can be further digested by prolidase to glycine and proline, and we found decreased glycylproline and increased glycine levels in urine collected from the LCG rats. Glycine is involved in the synthesis of collagen. Phenylacetylglycine is a glycine conjugate of phenylacetic acid. Decreased glycylproline and increased glycine and phenylacetylglycine levels in urine suggested disruption of bone collagen. The results of our study suggest that glycylproline, glycine, and phenylacetylglycine could be involved in the metabolic pathway of the degradation and biosynthesis of bone collagen, and that the low-calcium diet could have induced disruption of bone collagen. Ascorbic acid is necessary for the maintenance of connective tissue and bone, and it is an electron donor for enzymes involved in collagen hydroxylation [45,46]. D-Glucurono-6,3-lactone participates in the synthesis of ascorbate. The significant increase of D-glucurono-6,3-lactone levels in LCG urine indicates that the synthesis of vitamin C was reduced because of the lack of D-glucurono-6,3-lactone, and a decrease in ascorbic acid levels in the LCG urine was also seen. The decreased ascorbic acid and increased D-glucurono-6, 3-lactone levels could be an indication or a possible consequence, of impaired connective tissue and bone.

PTH induced the demineralization and inhibition of citrate excretion [47], and reduced citrate excretion was found in the LCG urine. Citrate has an important role in bone metabolism and bone diseases [48-50], and it can be considered a biomarker of calcium deficiency. Citric acid (citrate) can be formed in the Krebs cycle. Oxoglutaric acid and acetoacetic acid are the important biological compounds and are key intermediates in the Krebs cycle, therefore, the decreased oxoglutaric acid, acetoacetic acid, and citrate levels found in LCG urine suggest that the Krebs cycle could be impaired by calcium deficiency.

Xanthosine and uric acid are involved in purine metabolism. The increased xanthosine and decreased uric acid levels suggest that purine metabolism was possibly related to calcium metabolism. N-acetyl-l-tyrosine and L-glutamic acid are amino acids. The alterations of these two metabolites in our study suggest that calcium deficiency could lead to the abnormal metabolism of amino acid. Sebacic acid belongs to the dicarboxylic acid, which is involved in the metabolism of lipids and carbohydrates. Previous reports have documented that the medium-chain dicarboxylic acids (such as sebacic acid) are naturally occurring substances formed by cytochrome P450-mediated *ω*-oxidation of fatty acids in the cytosol of cells [51]. The decreased excretion of sebacic acid in rat urine suggests that calcium deficiency could impair *ω*-oxidation of medium-chain monocarboxylic acids. Taurine is a facilitator in the transport of calcium ions [52,53] and influences bone metabolism [54]. The decreased taurine levels in the LCG urine indicate impairment of bone metabolism. In earlier studies, it was found that indoxyl sulfate could impair osteoblast function and induce abnormal bone turnover [55,56]. Our results suggest that osteoblast function and bone turnover of the LCG rats could be impaired by increased indoxyl sulfate.

An animal urinary metabonomics study can provide continuous time-dependent data to allow assessment of the dynamic metabolic alterations and reveal the time-dependent pathophysiological changes. For instance, significantly increased levels of pyrophosphate, cAMP, glycine, indoxyl sulfate, and xanthosine, and decreased levels of citrate, oxoglutaric acid, acetoacetic acid, sebacic acid, and ascorbic acid were seen in the LCG rats in week 2, indicating that a 2-week low-calcium diet could result in metabolic changes in calcium and phosphorus levels, and in mild disruption of bone, bone collagen and bone turnover, and osteoblasts. This result is consistent with the results of the calcium-balance study, which could further explain the reason for the decrease in calcium excretion in urine and feces and the increase in calcium absorptivity. The peak area of pyrophosphate was decreased and that of ascorbic acid was increased persistently during weeks 1 to 12 (Figure 6A1, 6A2), indicating that body calcium homeostasis was gradually disrupted as the calcium deficiency progressed. Moreover, pseudouridine, naphthoxyacetic acid, cholanoic acid, and monomethyl fumarate in the LCG rats appeared to be changed significantly after week 6. At this stage, there was significantly perturbed expression of phosphate, amino acids, organic acids, fatty acids and purine metabolism, and disruption of bone, bone collagen, osteoblasts, and the Krebs cycle in the LCG rats as measured by the urinary metabonomics study. These results suggest that the normal patterns of bone resorption and remodeling in the LCG rats was impaired, thus abnormal bone resorption and remodeling had led to disorders of bone metabolism and decreased BMD (Figure 9B). Most of the identified biomarkers supported the molecular mechanisms and pathophysiological changes associated with calcium deficiency that have been reported previously. In the current study, we found, for the first time to our knowledge, that purine metabolism and the Krebs cycle are possibly related to calcium deficiency. It is possible that new metabolic pathways and unknown mechanisms are involved in the progression of calcium deficiency. Further research focused on these biomarkers is needed.

Because they allow greater control of conditions to reduce extrinsic variability, animal models have advantages over human studies when investigating biomarkers and elucidating subtle metabolite alterations. Thus, we firstly identified the calcium-deficient biomarkers from animal models, before verifying the potential application of three biomarkers in humans. Significant correlations were found between dietary calcium intakes and two of the biomarkers (pseudouridine and citrate). Because we did not control for confounding factors, such as gender, age, health state, disease, and the serum levels of 25(OH)VD_3 _and PTH, the true correlations might have been concealed to some extent.

There were some limitations to our studies. We used only male rats in the animal study, thus, the gender may have been a confounder in the metabolomics experiments. Moreover, the human study in post-menopausal women regarding the correlations between calcium intake and two biomarkers provides only a potential application of these two biomarkers in humans. Large, well-designed studies (including male and female subjects) that control for confounding factors are needed to verify in humans the potential application of these two biomarkers from our animal models.

## Conclusions

We have identified for the first time reliable biomarkers of calcium deficiency in male rats, by using a time-course analysis of discriminating metabolites in a low-calcium diet experiment, then repeating the low-calcium diet experiment and performing a calcium-supplement experiment. The identified biomarkers give new insights into the pathophysiological changes and molecular mechanisms of calcium deficiency. The correlations between calcium intake and two of the biomarkers provide the potential for further assessment and elucidation of the metabolic responses of calcium deficiency in humans. After further verification of these two biomarkers in large population, we anticipate that these two biomarkers might be useful for population screening and clinical diagnosis of calcium deficiency in the future.

## Abbreviations

BPI: baseline peak intensity; CSG: calcium-supplementation group; ESI: electrospray ionization; LCG: low-calcium group; NCG: normal-calcium group; OPLS-DA: orthogonal projection to latent structures discriminate analysis; PCA: principal component analysis; PLS-DA: partial least-squares discriminant analysis; UPLC/Q-TOF MS/MS: Ultra-performance liquid chromatography and quadrupole time-of-flight tandem mass spectrometry.

## Competing interests

The authors declare that they have no competing interests.

## Authors' contributions

WMQ, LY, and SCH conceived and designed the study. YX, NH, LR, and SY carried out the animal experiments. HYF and LLY carried out the UPLC/Q-TOF MSMS data collection. PHZH and NLX carried out data analysis and interpretation. FLJ performed the statistical analysis. ZHQJ and NH performed the human study. WMQ and WF drafted the manuscript. WF, LY, and SCH revised the manuscript content. All authors read and approved the final manuscript.

## Pre-publication history

The pre-publication history for this paper can be accessed here:

http://www.biomedcentral.com/1741-7015/11/86/prepub

## Supplementary Material

Additional file 1 **Ingredients of diet for the rats in animal experiments**. Click here for file

Additional file 2 **Results of the calcium metabolic-balance study between the normal-calcium group (NCG) and the low-calcium group (LCG)**. **(A) **urine calcium excretion; **(B) **fecal calcium excretion; **(C) **calcium intake; **(D) **calcium retention; **(E) **apparent absorptivity of calcium. Click here for file

Additional file 3 **Two-dimensional principal component analysis (PCA) score plots of urine samples (circle) and quality control (QC) samples (red cross) at weeks 1 to 12 in electrospray ionization (ESI) negative ion mode**. Comp, component; t[1], component 1; t[2], component 2. Click here for file

Additional file 4 **Permutation test result of the partial least-squares discriminant analysis (PLS-DA) model**. The R2Y value represents the goodness of fit of the model, and the Q2 value represents the predictability of the models. Click here for file

Additional file 5 **List of discriminating variables between the low-calcium group and normal-calcium group in animal experiment I**. RT, retention time. Click here for file

Additional file 6 **Urinary metabolic-profiling analysis of experiment II (repeated low-calcium diet experiment)**. **(A) **Principal component analysis (PCA) score plots of the low-calcium group (LCG; black triangles) and normal-calcium group (NCG; red circles) rats. (n = 24, week 1 to 12). Each data point represents one subject. **(B) **Partial least-squares discriminant analysis (PLS-DA) score plot between LCG (black triangles) and NCG (red circles). (n = 24, week 1 to 12). Each data point represents one subject. **(C) **Permutation test result of PLS-DA model. The R2Y value represents the goodness of fit of the model, and the Q2 value represents the predictability of the models. **(D) **Batch PLS score plot of urine samples mapped against time (n = 24, week 1 to 12). Dashed horizontal lines show two and three standard deviations for the dataset. LCG rats are shown as black lines and NCG rats as red lines. **(E) **PCA scores plots of LCG (black squares) and NCG (red triangles) rats. (A-C) Week 2, n = 24 (for two components R2Y = 48.8%, Q2 = 20.7%); week 4, n = 24 (for three components R2Y = 55.1%, Q2 = 26.4%) and week 9, n = 24 (for three components R2Y = 59.1%, Q2 = 28.7%). Each data point represents one subject. **(F) **Metabolic trajectory scores plots by PCA model derived from the urine of LCG rats (n = 12) from weeks 1 to 12. Black triangle: weeks 1 to 3; red star: weeks 4 to 8; blue circle: weeks 9 to 12. Comp, component. t[1], component 1; t[2], component 2. Click here for file

Additional file 7 **List of discriminating variables between the low-calcium diet group and normal-calcium diet group in animal experiment II (repeated low-calcium diet experiment). RT, retnation time**. Click here for file

Additional file 8 **Detailed identifying experiments using the quadrupole time-of-flight tandem mass spectrometry (UPLC/Q-TOF MS/MS) and authentic standards to elucidate the elemental composition of cAMP**. Click here for file

Additional file 9 **Identification data of eight biomarkers**. Click here for file
