# Supplementary material for: Calcium-deficiency assessment and biomarker identification by an integrated urinary metabonomics analysis
Source: BMC Med. 2013 Mar 28;11:86. doi: 10.1186/1741-7015-11-86 (PMC3652781; doi:10.1186/1741-7015-11-86)
Supplement: Additional file 1 — Ingredients of diet for the rats in animal experiments. [file 1741-7015-11-86-S1.DOC]

**Additional file 1:** Ingredients of diet feeding for the rats in animal experiments

| Composition | Amount (g/kg diet) | | |
| --- | --- | --- | --- |
|  | Normal calcium | Low calcium | High calcium |
| Ingredient | 0.50% Ca | 0.15% Ca | 1.5% Ca |
| Cornstarch | 397.486 | 397.486 | 397.486 |
| Casein (=85% Protein) | 200.000 | 200.000 | 200.000 |
| Dextrinized cornstarch | 132.000 | 132.000 | 132.000 |
| Sucrose | 100.000 | 100.000 | 100.000 |
| Soybean oil (alpha cellulose) | 70.000 | 70.000 | 70.000 |
| Fiber | 50.000 | 50.000 | 50.000 |
| Mineral mix | 35.000 | 35.000 | 35.000 |
| Vitamin mix | 10.000 | 10.000 | 10.000 |
| L-Cystine | 3.000 | 3.000 | 3.000 |
| Choline bitartrate (41.1%) | 2.500 | 2.500 | 2.500 |
| Tert-butylhydroquinone | 0.014 | 0.014 | 0.014 |
