# Supplementary material for: Calcium-deficiency assessment and biomarker identification by an integrated urinary metabonomics analysis
Source: BMC Med. 2013 Mar 28;11:86. doi: 10.1186/1741-7015-11-86 (PMC3652781; doi:10.1186/1741-7015-11-86)
Supplement: Additional file 2 — Results of the calcium metabolic-balance study between the normal-calcium group (NCG) and the low-calcium group (LCG). (A) urine calcium excretion; (B) fecal calcium excretion; (C) calcium intake; (D) calcium retention; (E) apparent absorptivity of calcium. [file 1741-7015-11-86-S2.DOC]

**Additional file 2:** Results of the calcium metabolic balance study between normal and low calcium diet groups: A: urine calcium excretion; B: fecal calcium excretion; C: calcium intake; D: calcium retention; E: calcium apparent absorptivity.


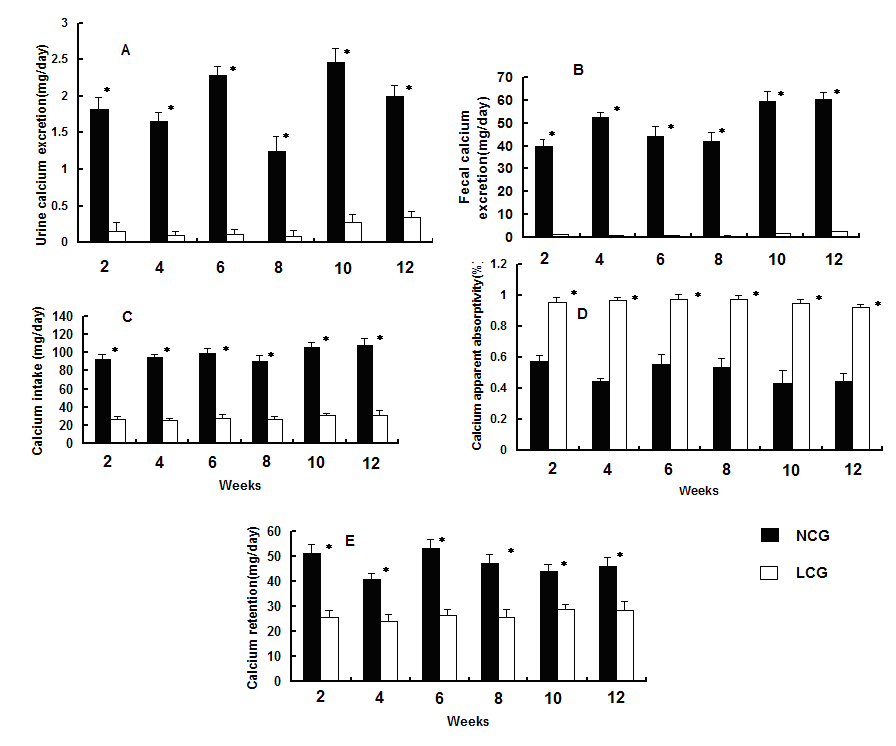


*Statistically significant differences (*p*<0.05) for mean values compared with low calcium

diet group at same time points.

**
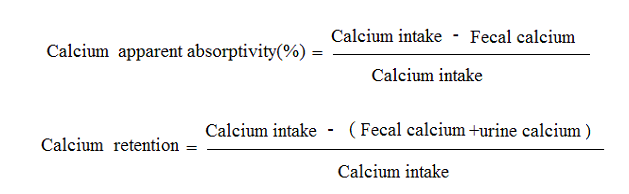
**
