# Supplementary material for: Calcium-deficiency assessment and biomarker identification by an integrated urinary metabonomics analysis
Source: BMC Med. 2013 Mar 28;11:86. doi: 10.1186/1741-7015-11-86 (PMC3652781; doi:10.1186/1741-7015-11-86)
Supplement: Additional file 3 — Two-dimensional principal component analysis (PCA) score plots of urine samples (circle) and quality control (QC) samples (red cross) at weeks 1 to 12 in electrospray ionization (ESI) negative ion mode. Comp, component; t[1], component 1; t[2], component 2. [file 1741-7015-11-86-S3.DOC]

**Additional file 3:** Two-dimensional PCA scores plots of urine samples (black circles) and QC samples (red crosses) of week 1-12 in ESI negative ion mode.


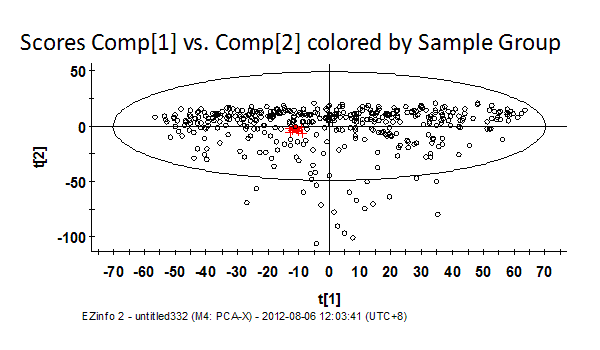


Experiment **I** 384 samples (LCG and NCG, week 1-12).

Experiment **II** 288 samples (**repeated low-calcium diet experiment:** LCG and NCG, week 1-12).

Experiment **II** 180 samples (**calcium supplement experiment:** LCG, NCG and CSG, week 8-12).

**Table Reproducibility of method from 4 ions of the quality control sample in the negative ESI modes (n = 6)**

1. Experiment **I**

| **m/z** | **RT** | | | **Peak Intensity** | | |
| --- | --- | --- | --- | --- | --- | --- |
|  | **RT** | **SD** | **RSD** | **INT** | **SD** | **RSD** |
| 223.9991 | 0.56 | 0.004 | 0.0071 | 3453 | 139 | 0.04 |
| 297.148 | 3.18 | 0 | 0 | 17150 | 137 | 0.008 |
| 105.0282 | 4.855 | 0.01 | 0.002 | 8731 | 172 | 0.019 |
| 353.2567 | 8.905 | 0.005 | 0.0006 | 4106 | 107 | 0.026 |

2. Experiment **II**

| **m/z** | **RT** | | | **Peak Intensity** | | |
| --- | --- | --- | --- | --- | --- | --- |
|  | **RT** | **SD** | **RSD** | **INT** | **SD** | **RSD** |
| 223.9991 | 0.53 | 0.004 | 0.0073 | 3641 | 118 | 0.032 |
| 297.148 | 3.16 | 0 | 0 | 16010 | 143 | 0.009 |
| 105.0282 | 4.84 | 0 | 0 | 8783 | 152 | 0.017 |
| 353.2567 | 8.93 | 0.005 | 0.00056 | 4510 | 130 | 0.0288 |

3. Experiment **I and II**

| **m/z** | **RT** | | | **Peak Intensity** | | |
| --- | --- | --- | --- | --- | --- | --- |
|  | **RT** | **SD** | **RSD** | **INT** | **SD** | **RSD** |
| 223.9991 | 0.545 | 0.015 | 0.027 | 3560 | 220 | 0.062 |
| 297.148 | 3.17 | 0.003 | 0.001 | 16550 | 254 | 0.015 |
| 105.0282 | 4.847 | 0.008 | 0.002 | 8745 | 191 | 0.0218 |
| 353.2567 | 8.92 | 0.189 | 0.021 | 4306 | 217 | 0.05 |
