# Supplementary material for: Calcium-deficiency assessment and biomarker identification by an integrated urinary metabonomics analysis
Source: BMC Med. 2013 Mar 28;11:86. doi: 10.1186/1741-7015-11-86 (PMC3652781; doi:10.1186/1741-7015-11-86)
Supplement: Additional file 4 — Permutation test result of the partial least-squares discriminant analysis (PLS-DA) model. The R2Y value represents the goodness of fit of the model, and the Q2 value represents the predictability of the models. [file 1741-7015-11-86-S4.DOC]

**Additional file 4:** Permutation test result of the PLS-DA model. The *R*^2^*Y* value represents the goodness of fit of the model. The *Q*^2^ value represents the predictability of the models.


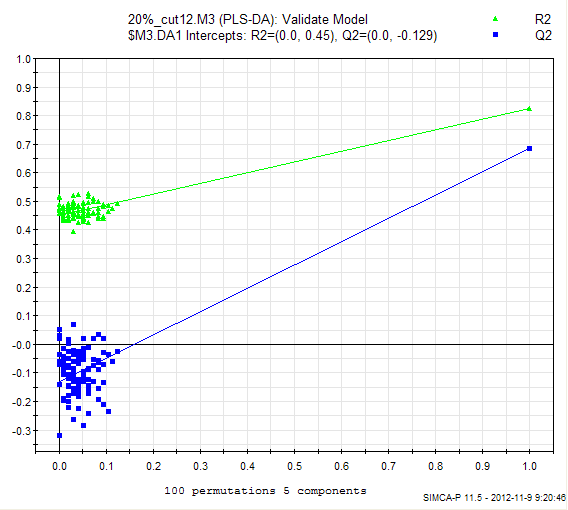


The first five components of PLS-DA model: R2X =0.826, Q2=0.687. The idea of this validation is to compare the goodness of fit (*R^2^* and *Q^2^*) of the original model with the goodness of fit of several models based on data where the order of the Y-observations have been randomly permuted, while the X-matrix has been kept intact. The values of *R^2^* and *Q^2^* for the original model and the Y-permuted models were in the top right corner and the bottom-left corner of the plot, respectively.
