# Supplementary material for: Calcium-deficiency assessment and biomarker identification by an integrated urinary metabonomics analysis
Source: BMC Med. 2013 Mar 28;11:86. doi: 10.1186/1741-7015-11-86 (PMC3652781; doi:10.1186/1741-7015-11-86)
Supplement: Additional file 5 — List of discriminating variables between the low-calcium group and normal-calcium group in animal experiment I. RT, retention time. [file 1741-7015-11-86-S5.DOC]

**Additional file 5:** List of discriminating variables between low calcium group and normal calcium group in animal experiment I.

| No | RT | Actual mass |  | No | RT | Actual mass |
| --- | --- | --- | --- | --- | --- | --- |
| 1 | 1.2 | 191.0173 |  | 25 | 4.31 | 308.1174 |
| 2 | 1.20 | 111.0073 |  | 26 | 1.08 | 129.0170 |
| 3 | 0.67 | 176.9366 |  | 27 | **1.16** | **67.0198** |
| 4 | 0.98 | 145.0158 |  | **28** | **9.04** | **221.0811** |
| 5 | 0.96 | 175.0251 |  | **29** | **6.81** | **187.0034** |
| 6 | 5.89 | 192.0629 |  | **30** | **1.19** | **87.0045** |
| 7 | 5.97 | 74.0238 |  | **31** | **4.88** | **211.9939** |
| 8 | 1.02 | 101.0237 |  | **32** | **6.81** | **231.0723** |
| 9 | 6.42 | 283.0714 |  | **33** | **3.62** | **194.0459** |
| 10 | 6.81 | 231.0723 |  | **34** | **8.65** | **343.0807** |
| 11 | 9.30 | 407.2809 |  | **35** | **7.54** | **187.0106** |
| 12 | 1.14 | 167.0211 |  |  |  |  |
| 13 | 0.99 | 293.0520 |  |  |  |  |
| 14 | 1.02 | 243.0625 |  |  |  |  |
| 15 | 8.19 | 201.1134 |  |  |  |  |
| 16 | 6.44 | 113.0231 |  |  |  |  |
| 17 | 0.91 | 124.0059 |  |  |  |  |
| 18 | 8.21 | 199.1312 |  |  |  |  |
| 19 | 6.43 | 212.0020 |  |  |  |  |
| 20 | 6.44 | 175.0236 |  |  |  |  |
| 21 | 7.15 | 171.1008 |  |  |  |  |
| 22 | 5.87 | 222.0808 |  |  |  |  |
| 23 | 8.46 | 113.0964 |  |  |  |  |
| 24 | 2.36 | 328.0451 |  |  |  |  |
