# Supplementary material for: Calcium-deficiency assessment and biomarker identification by an integrated urinary metabonomics analysis
Source: BMC Med. 2013 Mar 28;11:86. doi: 10.1186/1741-7015-11-86 (PMC3652781; doi:10.1186/1741-7015-11-86)

**Additional file 6:** **(A)** PCA scores plots of the LCG and NCG rats. (n=24, week 1-12). Red circle: normal calcium diet rats. Black triangle: low calcium diet rats. A single data point represents one subject.

**(B)** PLS-DA scores plot between LCG and NCG. (n=24, week 1-12). Red circle: normal calcium diet rats. Black triangle: low calcium diet rats. A single data point represents one subject.

**(C)** Permutation test result of PLS-DA model. The *R*^2^*Y* value represents the goodness of fit of the model. The *Q*^2^ value represents the predictability of the models.

**(D)** Batch PLS scores plot of urine samples mapped with time (n=24, week 1-12). Dashed horizontal lines show two and three standard deviations for the data set. Black lines represent NCG; red lines represent LCG.

**(E)** PCA scores plots of normal calcium and low calcium diet rats. Red triangle: normal calcium diet rats. Black box: low calcium diet rats. A, B and C: week 2, n=24 (for the first two components *R*2*Y* =48.8%, *Q*2 =20.7%); week 4, n=24 (for the first three components *R*2*Y* =55.1%, *Q*2 =26.4%) and week 9, n=24 (for the first three components *R*2*Y* =59.1%, *Q*2 =28.7%). A single data point represents one subject.

**(F)** Metabolic trajectory scores plots by PCA model derived from the urine of low calcium diet rats (n=12) from weeks 1-12. Black triangle: weeks 1-3; red star: weeks 4-8; blue circle: weeks 9-12.

**
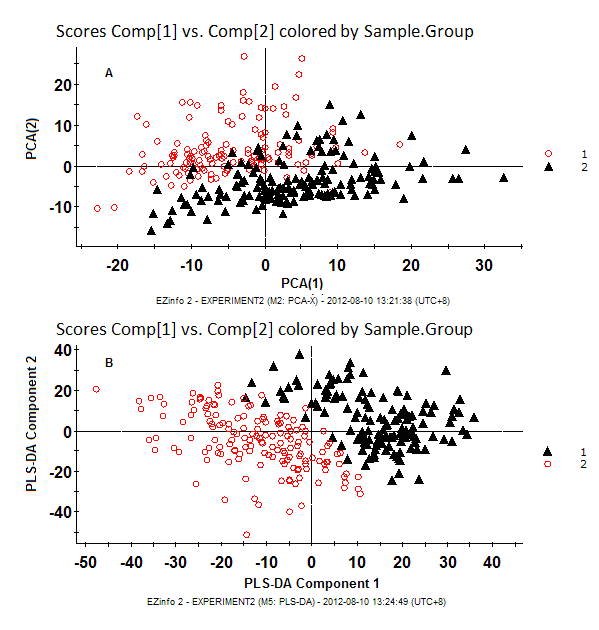
**


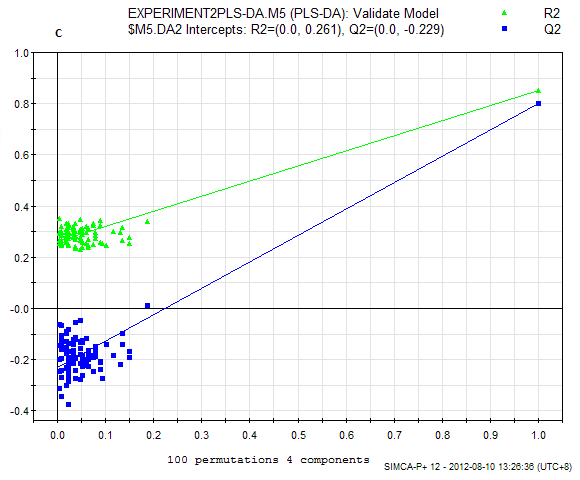


**
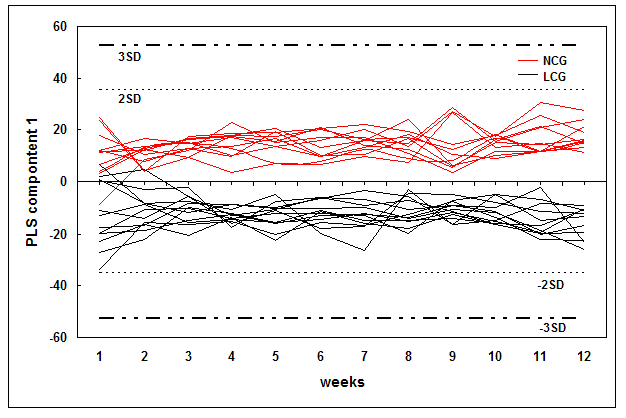
**


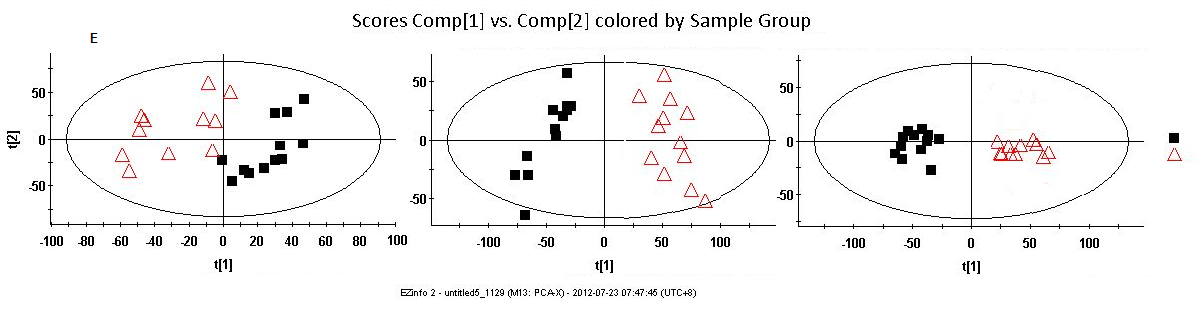


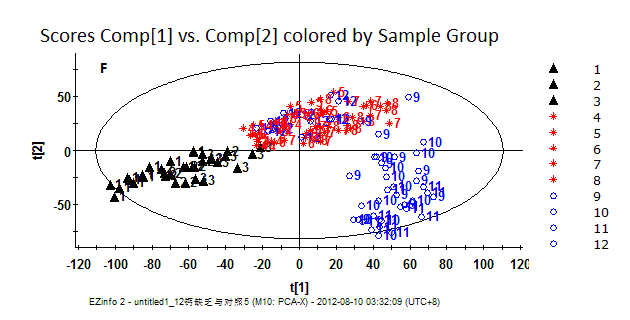

Supplement: Additional file 6 — Urinary metabolic-profiling analysis of experiment II (repeated low-calcium diet experiment). (A) Principal component analysis (PCA) score plots of the low-calcium group (LCG; black triangles) and normal-calcium group (NCG; red circles) rats. (n = 24, week 1 to 12). Each data point represents one subject. (B) Partial least-squares discriminant analysis (PLS-DA) score plot between LCG (black triangles) and NCG (red circles). (n = 24, week 1 to 12). Each data point represents one subject. (C) Permutation test result of PLS-DA model. The R2Y value represents the goodness of fit of the model, and the Q2 value represents the predictability of the models. (D) Batch PLS score plot of urine samples mapped against time (n = 24, week 1 to 12). Dashed horizontal lines show two and three standard deviations for the dataset. LCG rats are shown as black lines and NCG rats as red lines. (E) PCA scores plots of LCG (black squares) and NCG (red triangles) rats. (A-C) Week 2, n = 24 (for two components R2Y = 48.8%, Q2 = 20.7%); week 4, n = 24 (for three components R2Y = 55.1%, Q2 = 26.4%) and week 9, n = 24 (for three components R2Y = 59.1%, Q2 = 28.7%). Each data point represents one subject. (F) Metabolic trajectory scores plots by PCA model derived from the urine of LCG rats (n = 12) from weeks 1 to 12. Black triangle: weeks 1 to 3; red star: weeks 4 to 8; blue circle: weeks 9 to 12. Comp, component. t[1], component 1; t[2], component 2. [file 1741-7015-11-86-S6.DOC]
