# Supplementary material for: Calcium-deficiency assessment and biomarker identification by an integrated urinary metabonomics analysis
Source: BMC Med. 2013 Mar 28;11:86. doi: 10.1186/1741-7015-11-86 (PMC3652781; doi:10.1186/1741-7015-11-86)
Supplement: Additional file 8 — Detailed identifying experiments using the quadrupole time-of-flight tandem mass spectrometry (UPLC/Q-TOF MS/MS) and authentic standards to elucidate the elemental composition of cAMP. [file 1741-7015-11-86-S8.DOC]

**Additional file 8:** Detailed identifying experiments utilizing the tandem Q/TOF MS/MS and authentic standards to elucidate the elemental composition of cAMP

First, the probable empirical formulae of the ion of *m/z* 328.0475 was preliminary derived on the basis of accurate mass measurement (a mass error < 20 ppm) and considering the relative intensities of the isotope peaks through the high-resolution MS spectra. The elemental composition was **C_10_H_12_N_5_O_6_P** and the PPM was 8.5 ppm**.** After searching databases on line, we concluded that the **C_10_H_12_N_5_O_6_P** might be **Cyclic AMP, Adenosine 2',3'-cyclic phosphate, 3',5'-Cyclic dGMP or 3',5'-Cyclic AMP.**

**Secondly,** as shown in Figure 1 and 2, **compared with the MSMS spectum of Cyclic** **AMP** , the ion of *m/z* 328.0475 had more possibility to be **Cyclic AMP.**

Thirdly, in order to further verify the elemental composition, the authentic standard **of Cyclic AMP (**purchased from Sigma–Aldrich (St Louis, MO, USA)) **and urine sample were analyzed by UPLC/Q-TOF MS.** MS/MS spectra were obtained by Q-TOF Mass. As shown in Figure 3 and 4, the ion of *m/z* 328.0475 exhibited the same retention time, *m/z* and MSMS spectra as the standard **of Cyclic AMP**, the ion at 2.38 min in rat urine sample was confirmed as **Cyclic AMP**.


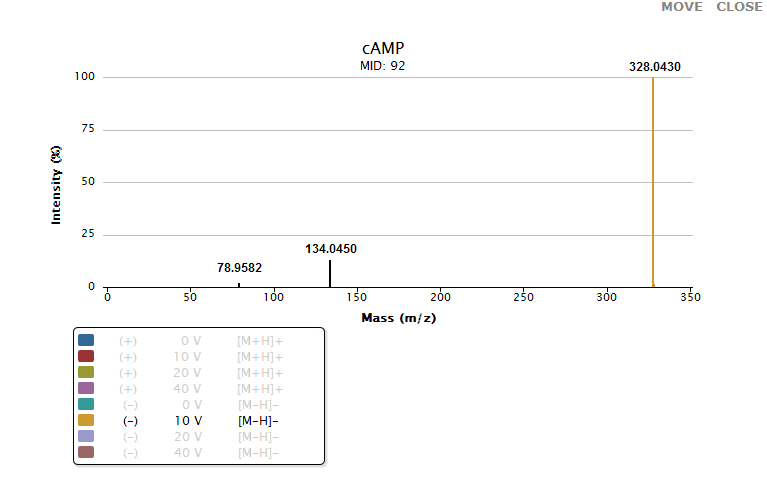


**Figure 1** MSMS spectra of c-AMP from METLIN MASS database.


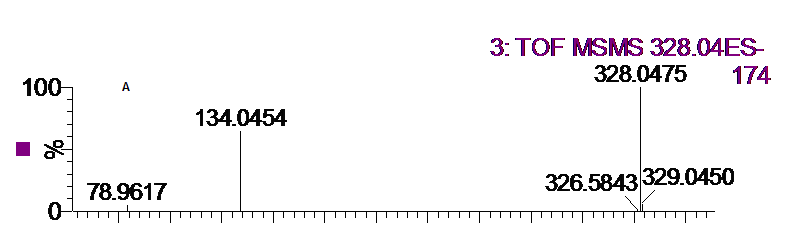


**Figure 2** MSMS spectra of the ion of *m/z* 328.0475 from urine sample.


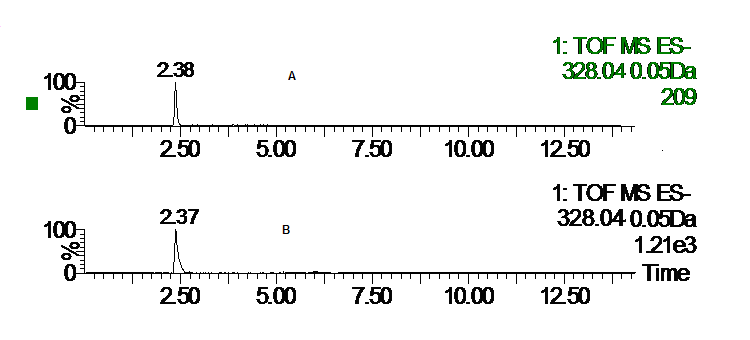


**Figure 3** The chromatograms for the retention times of *m/z* 328.04 ion from urine sample (A) and c-AMP standard solution (B)


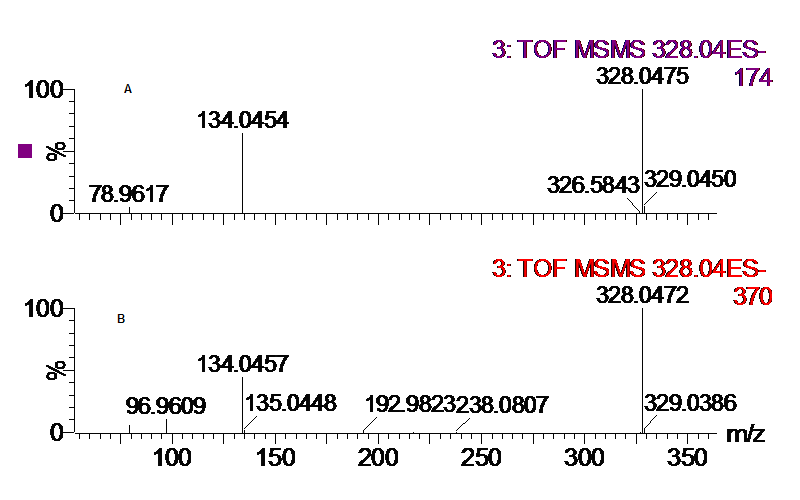


**Figure 4** MSMS spectra of *m/z* 328.04 ion from urine sample (A) and c-AMP standard solution (B)
