# Supplementary material for: Calcium-deficiency assessment and biomarker identification by an integrated urinary metabonomics analysis
Source: BMC Med. 2013 Mar 28;11:86. doi: 10.1186/1741-7015-11-86 (PMC3652781; doi:10.1186/1741-7015-11-86)
Supplement: Supplementary file 9 [file 1741-7015-11-86-S9.DOC]

Additional file 9: Identification data of 8 biomarkers
1. pseudouridine
Figure 1 The chromatograms for the retention times of m/z 243.06 ion from urine sample (A) and pseudouridine standard solution (B)


Figure 2 MSMS spectra of m/z 243.06 ion from urine sample (A) and pseudouridine standard solution (B)





2. citrate

Figure 3 The chromatograms for the retention times of citrate standard solution (A) and m/z 191.01 ion from urine sample (B)

Figure 4 MSMS spectra of citrate standard solution (A) and m/z 191.01 ion from urine sample (B)






3. 4-Oxo-2-pentenoic acid（6.44，113.0231）：
95.0140
¬- (+0H)



95.0133 (+0.7.mDa) (S:0.5, B:1)
C5H3O2 (-H2O)

	85.0301
¬- (+5H)	


85.0290 (+1.1.mDa) (S:1.5, B:2)
C4H5O2 (-CO)

	67.0198
¬- (+0H)



67.0184 (+1.4.mDa) (S:1.0, B:1)
C4H3O (-CH2O2)

		57.0351
¬- (+5H)



57.0340 (+1.1.mDa) (S:1.5, B:2)
C3H5O (-C2O2)

				














4. Monomethyl fumarate(1.08, 129.0170):
	ID (job)	19			Mass (Da)	130.0266			Formula	C5H6O4			DBE	4		
113.9962
¬.- (+1H)



113.9953 (+0.9.mDa) (S:0.5, B:1)
C4H2O4 (-CH3)

	101.0253
¬- (+4H)



101.0239 (+1.4.mDa) (S:0.5, B:1)
C4H5O3 (-CO)

	85.0300
¬- (+2H)



85.0290 (+1.0.mDa) (S:1.0, B:1)
C4H5O2 (-CO2)

						73.0338
¬- (+4H)



73.0290 (+4.8.mDa) (S:1.0, B:1)
C3H5O2 (-C2O2)

	57.0288
¬- (+5H)



57.0340 (-5.2.mDa) (S:1.5, B:2)
C3H5O (-C2O3)

			














5. Naphthoxylactic acid ( 6.81, 231.0657):
231.0787
¬- (+1H)



231.0657 (+13.0.mDa) (S:8.0, B:0)
C13H11O4 (-none)

	187.0805
¬- (+2H)



187.0759 (+4.6.mDa) (S:1.0, B:1)
C12H11O2 (-CO2)

	156.0453
¬.- (+1H)



156.0575 (-12.2.mDa) (S:1.0, B:1)
C11H8O (-C2H3O3)

		128.0454
¬.- (+3H)



128.0626 (-17.2.mDa) (S:0.5, B:1)
C10H8 (-C3H3O4)

	74.0281
¬.- (+1H)



74.0004 (+27.7.mDa) (S:1.0, B:1)
C2H2O3 (-C11H9O)

			

6. N-[(3,5-dimethyl-2,3-dihydro-1,2-oxazol-3-yl)sulfonyl]aspartic acid (0.99, 293.0520):

293.0527
¬- (+1H)



293.0443 (+8.3.mDa) (S:1.0, B:0)
C9H13N2O7S (-none)

	275.0430
¬- (+0H)



275.0338 (+9.2.mDa) (S:0.5, B:1)
C9H11N2O6S (-H2O)

	203.0551
¬- (+1H)



203.0490 (+6.1.mDa) (S:2.0, B:2)
C7H11N2O3S (-C2H2O4)

		195.8945
¬- (+2H)



195.9916 (-97.1.mDa) (S:0.5, B:1)
C4H6NO6S (-C5H7NO)

	194.9312
¬.- (+1H)



194.9838 (-52.6.mDa) (S:0.5, B:1)
C4H5NO6S (-C5H8NO)

	145.0123
¬.- (+0H)



144.9834 (+28.9.mDa) (S:2.0, B:2)
C4H3NO3S (-C5H10NO4)

		131.0294
¬.- (+1H)



131.0219 (+7.5.mDa) (S:1.0, B:1)
C4H5NO4 (-C5H8NO3S)

	96.9587
¬- (-1H)



96.9926 (-33.9.mDa) (S:1.0, B:2)
C4HO3 (-C5H12N2O4S)

	96.9587
¬.- (+1H)



97.0528 (-94.1.mDa) (S:0.5, B:1)
C5H7NO (-C4H6NO6S)

						




7. Ethyl isobutyl ketone(8.46, 113.0964 ):
R85.0461
¬- (+2H)



85.0653 (-19.2.mDa) (S:1.0, B:1)
C5H9O (-C2H4)

	69.0416
¬- (+0H)



69.0340 (+7.6.mDa) (S:1.0, B:1)
C4H5O (-C3H8)

	57.0355
¬- (+2H)



57.0340 (+1.5.mDa) (S:1.0, B:1)
C3H5O (-C4H8)

						








8. 6-(2-hydroxycyclopentyl) hexanoic acid (8.21, 199.1352):
199.1352
¬- (+1H)



199.1334 (+1.8.mDa) (S:2.0, B:0)
C11H19O3 (-none)

	181.1177
¬- (+0H)



181.1229 (-5.2.mDa) (S:0.5, B:1)
C11H17O2 (-H2O)

	156.1406
¬.- (+3H)



156.1514 (-10.8.mDa) (S:1.0, B:1)
C10H20O (-CO2)

		139.1129
¬- (+0H)



139.1123 (+0.6.mDa) (S:1.0, B:1)
C9H15O (-C2H4O2)

	127.1111
¬- (+2H)



127.1123 (-1.2.mDa) (S:1.0, B:1)
C8H15O (-C3H4O2)

	121.0662
¬- (-1H)



121.1017 (-35.5.mDa) (S:1.5, B:2)
C9H13 (-C2H6O3)

		111.0834
¬- (+0H)



111.0810 (+2.4.mDa) (S:1.0, B:1)
C7H11O (-C4H8O2)

	85.0671
¬- (+2H)



85.0653 (+1.8.mDa) (S:1.0, B:1)
C5H9O (-C6H10O2)

	74.0255
¬.- (+3H)



74.0368 (-11.3.mDa) (S:1.0, B:1)
C3H6O2 (-C8H13O)

		71.0274
¬- (+0H)



71.0133 (+14.1.mDa) (S:1.0, B:1)
C3H3O2 (-C8H16O)
